# Prevalence of chlamydia and gonorreheae among transgender women and *travestis* in five Brazilian capitals, 2019–2021

**DOI:** 10.1590/1980-549720240006.supl.1

**Published:** 2024-08-19

**Authors:** Katia Cristina Bassichetto, Sandro Sperandei, Daniel Jason McCartney, Carla Gianna Luppi, Roberto José Carvalho da Silva, Sandra Araújo, Laio Magno, Maria Luíza Bazzo, Gwenda Hughes, Philippe Mayaud, Inês Dourado, Maria Amélia de Sousa Mascena Veras

**Affiliations:** I Santa Casa de São Paulo, School of Medical Sciences – São Paulo (SP), Brazil.; II Western Sydney University, Translational Health Research Institute – Sydney, Australia.; III London School of Hygiene & Tropical Medicine, Faculty of Infectious & Tropical Diseases, Department of Clinical Research – London, United Kingdom.; IV State Health Secretariat of São Paulo, STD/AIDS Reference and Training Center – São Paulo (SP), Brazil.; V Universidade do Estado da Bahia, Department of Life Sciences – Salvador (BA), Brazil.; VI Universidade Federal de Santa Catarina, Postgraduate Program in Pharmacy/Health Sciences Center, Laboratory of Molecular Biology, Microbiology and Serology – Florianópolis (SC), Brazil.; VII Universidade Federal da Bahia, Institute of Public Health – Salvador (BA), Brazil.

**Keywords:** Epidemiology, Sexually transmitted diseases, Transgender persons, Travestis, Chlamydia, Gonorrhea

## Abstract

**Objective:**

To estimate the prevalence and factors associated with the detection of *Chlamydia trachomatis* (CT) and *Neisseria gonorrhoeae* (NG) in transgender women and *travestis* in five Brazilian capitals.

**Methods:**

Data were obtained from a cross-sectional study conducted between 2019 and 2021, with participants recruited through Respondent Driven Sampling in São Paulo, Campo Grande, Manaus, Porto Alegre and Salvador. Detection of CT and NG was analyzed at three collection sites (anorectal, oropharyngeal and urethral). Mixed logistic regression models were employed to identify associated factors.

**Results:**

A total of 1,297 recruited participants provided biological material to detect these infections. The prevalences of CT, NG and coinfection were 11.5%, 13.3% and 3.6%, respectively. Independent associations with CT infections included past (OR=1.73; 95%CI 1.02–2.95), current (OR=2.13; 95%CI 1.23–3.69), and part-time sex work (OR=2.75; 95%CI 1.60–4.75), as well as lifetime injectable drug use (OR=3.54; 95%CI 1.49–8.40). For NG, associations were observed with lifetime injectable drug use (OR=1.91; 95%CI 1.28–2.84) and sexual orientation, including heterosexual (OR=3.44; 95%CI 1.35–8.82), homosexual (OR=5.49; 95%CI 1.89–15.97), and bisexual (OR=3.21; 95%CI 1.06–9.68). Coinfection was associated with use of illicit drugs in the last 12 months (OR=2.34, 95%CI 1.10–5.00), and younger age was associated with all investigated outcomes.

**Conclusion:**

Estimated prevalences of CT, NG and co-infection were higher among transgender women and *travestis* compared to the general population, particularly among younger, individuals engaged in sex work and illicit drug use.

## INTRODUCTION

The World Health Organization (WHO) estimated approximately 374 million new sexually transmitted infections (STIs) in 2020. Among the most prevalent are *Chlamydia trachomatis* (CT) and *Neisseria gonorrhoeae* (NG), accounting for 128.5 million and 82.4 million cases, respectively, with 29.8 million and 13.8 million in the Americas alone. These infections pose a significant public health challenge and incur substantial costs^1^. The incidence of co-infection varies greatly across countries and among different population groups. However, there are few studies on the transgender population, a critical gap that hinders the development of targeted interventions and policies to address their specific needs^2-5^.

Prevention and control strategies for CT and NG are foreseen in global health initiatives. These efforts include the commitment to reduce the incidence of NG by 90% by 2030, focusing on populations most vulnerable to STIs^6^, and strengthening the Gonococcal Antimicrobial Surveillance Program (GASP)^7-9^.

The prevalence of these infections has been extensively investigated across various contexts, revealing high rates among men who have sex with men (MSM) and transgender women. Moreover, prevalence rates show considerable variability based on the anatomical site of infection^10,11^. More than half of extragenital infections are asymptomatic or oligosymptomatic, contributing to maintaining the chain of transmission^12^.

Several international public and sexual health institutions have recommended the availability of CT and NG screening for populations at the highest risk, including transgender individuals^6^.

In Brazil, implementing these strategies remain a challenge, especially due to the difficulty in expanding the detection of the etiological agent in the Brazilian Unified Health System (*Sistema Único de Saúde* – SUS), currently restricted to sentinel units. However, there are promising prospects for expansion^7,13,14^.

The Clinical Protocol and Therapeutic Guidelines (*Protocolo Clínico e Diretrizes Terapêuticas* – PCDT) for Comprehensive Care for People with STIs, prepared by the Ministry of Health (MoH)^15^, recommends biannual screening. This includes the collection of anal swabs to detect CT and NG for individuals engaging in receptive anal practices without barrier protection. The Ministry of Health has made efforts to facilitate the diagnosis and treatment of asymptomatic patients and their partners by providing tests to detect these pathogens, aligning with priorities for comprehensive care for people with STIs^15^. However, screening is not yet fully implemented into routine care and many extragenital infections remain undiagnosed, which can lead to complications such as chronic pain and proctitis^16^, and increasing the risk of HIV transmission^15^.

Several studies have investigated the prevalence of CT and NG in various populations: MSM^2,5,17-19^, users of STI clinics^3,4,20,21^, people with HIV^22,23^, parturients^24^, women receiving human reproduction services^16^, and sexually active young women^25^. However, when these studies involve the transgender population, they are often on reduced samples or restricted to specialized services^2,10,12,17,19,20^. It is crucial to estimate the prevalence of these infections in transgender women and *travestis*, especially by recruiting participants by peers rather than solely relying on specialized services.

The objective of this study was to estimate the prevalence and factors associated with CT, NG, and coinfections, according to anatomical site (anorectal, oropharyngeal, and urethral) and presence of signs and symptoms, in transgender women and *travestis*, in five Brazilian capitals.

## METHODS

### Design

Data from TransOdara, a cross-sectional study with quantitative and qualitative approach, carried out between 2019 and 2021, in five Brazilian capitals (São Paulo, Campo Grande, Manaus, Porto Alegre, and Salvador), were used to estimate the prevalence of eight STIs, including CT and NG. It also sought to demonstrate the feasibility of implementing point-of-care strategies to provide resources for prevention and treatment available in SUS (pre- and post-exposure prophylaxis for HIV and vaccines against hepatitis B and HPV) and/or treatments for STIs. These services were made available either on the same day of participation in the research or through referrals mediated by the researchers^26^.

Data collection was carried out at designated research locations and followed a structured flow. This process included confirming eligibility, offering laboratory tests, conducting interviews and administering socio-behavioral questionnaires, pre- and post-nursing consultation, medical consultation, including physical examination, regardless of the presence of signs and symptoms, and providing referrals when necessary. The instruments used during data collection included Pre-eligibility Forms, Acceptability of Pre-Consultation Collection and Pre-Consultation Procedures, Clinical Assessment and Follow-up, Acceptability of Collection and Post-Consultation Procedures, and Laboratory Assessment, in addition to the socio-behavioral questionnaire.

### Sample

The sampling technique employed was respondent driven sampling (RDS), suitable for recruiting difficult-to-reach populations^27^. A total of 1,317 participants were included in the study. Further information regarding recruitment strategies, eligibility criteria, sample calculation, and research flow can be found in the methodological article, within this supplement^26^.

### Prevalences of chlamydia and gonorrheae and selected variables

Urine samples, and rectal and oropharyngeal swabs were collected from participants, either by a healthcare professional or through self-collection, using the Multi-collect kit, according to the participant’s choice. Those who opted for self-collection received standardized guidance, including illustrative leaflets, and conducted the procedure at the research site.

The dependent variable was the detectable results for CT or NG, in at least one of the collection sites, as well as the presence of CT/NG co-infection. These variables were analyzed based on the cities where the research was carried out and the presence of signs and symptoms (only urethral discharge, only anal discharge, or both).

The samples collected in São Paulo (São Paulo STI/AIDS Reference and Treatment Center) were transported daily to the laboratory within the same unit where the research was carried out. Samples collected in the other capitals were sent air-shipped to São Paulo, packed in transport boxes with dry ice for analysis in the same laboratory. Upon arrival, they were kept in the Multi-collect kit and promptly stored in a freezer at -20°C, until processing. Analysis was conducted using Abbott’s M2000 SP/RT equipment. The samples were expected to remain stable for 90 days after collection.

The detection method for CT and NG was Polymerase Chain Reaction (PCR). The validity of the assay procedure and the qualitative determination of CT DNA plasmid and NG genomic DNA in swab samples were established using Abbott Real Time CT/NG Controls.

The independent variables analyzed for socioeconomic and demographic characterization were: age (18–25; 26–36; 37 years old or older); race/color (white; black/brown; other); religion (no religion; Afro-Brazilian; evangelical; Catholic); sexual orientation (pansexual; heterosexual; homosexual, gay or lesbian); marital status (married; in a relationship; single); education (elementary school, high school, and university); housing situation (own; rented; with friends/family; unstable); sex work (never; only in the past; current, partially; current as main activity); and the others variables categorized as “yes/no”: drug use in the last 12 months and lifetime use of injectable drugs, history of incarceration, history of aggression, consent in the first sexual intercourse, use of a condom in the first sexual intercourse, with the last steady partner and with the last casual partner.

#### Statistical analysis

A descriptive analysis was carried out for the explanatory variables, presenting absolute and relative frequencies. Univariate logistic models with mixed effects were constructed for the collection cities, and variables with a p-value less than or equal to 0.3 were selected for the construction of the final multivariate models^28^. To this end, the StatisticalModels package, the backward stepwise strategy, and the maximum likelihood criterion were used. RDS weights were not used as they did not offer advantages and could potentially lead to biased results^29^. The results of the logistic models were presented as odds ratios (OR) and 95% confidence intervals (95%CI). All analyses were performed in R, version 4.3.1^30^.

#### Ethical aspects

The project was approved by the Research Ethics Committee of Santa Casa de São Paulo (CAAE 05585518.7.0000.5479, opinion n°: 3.126.815 – January 30, 2019), as well as by other participating institutions. Informed consent was obtained from all participants included in the study^26^.

## RESULTS

In this study, biological materials (urine, oropharyngeal and/or anal swabs) for detection of CT and/or NG were provided by 98.5% of participants recruited in TransOdara (1,297/1,317) — Campo Grande (n=181), Manaus (n=335), Porto Alegre (n=189), Salvador (n=190), and São Paulo (n=402). The mean age of these participants was 32.5 years (standard deviation – SD=9.88). The majority (70%) self-reported their skin color as black/brown; 36.5% reported having no religion; 78.3% identified as heterosexual; 71.8% were single; and 70% had attended or completed high school. Approximately 85% of participants lived on low income — 43.8% received less than the minimum wage; 36.2% lived in rented housing and 21.3% reported sex work as their main occupation. Drug use in the last 12 months and injectable drug use at some point in life were reported, respectively, by 64.3% and 2.2% of participants; 22.7% had already been arrested and 50.3% had experienced sexual assault at some point in their lives; 14.2% reported that their first sexual intercourse was forced; and in the last 6 months, 31% and 16.3% did not use a condom with their last steady partner or with their commercial partner, respectively.

The prevalence of these infections, according to the cities, is shown in Table 1. Of the 1,297 participants, 11.5% (95%CI 9.8–13.2) tested positive for CT, 13.3% (95%CI 11.5–15.1) for NG, and 3.6% (95%CI 2.6–4.6) for co-infection. Regardless of the type of material collected, the prevalence for CT ranged between 15.3% (95%CI 10.1–20.5) (Salvador) and 8.8% (95%CI 4.6–13.0) (Campo Grande); for NG, it varied between 19.7% (95%CI 15.4–24.0) (Manaus) and 8.5% (95%CI 5.8–11.2) (São Paulo); and for co-infections, between 6.3% (95%CI 2.8–9.8) (Salvador) and 2.1% (95%CI 0.0–4.2) (Porto Alegre), with no statistically significant difference observed.

**Table 1:** Prevalence estimates (n, %, and 95%CI) of chlamydia, gonorrhea, and co-infection, among transgender women and *travestis*, in five Brazilian capitals. TransOdara Study, 2019–2021.

Municipalities	Chlamydia			Gonorrhea			Coinfection			n
	n	%	95%CI	n	%	95%CI	n	%	95%CI	
Campo Grande	16	8.8	(4.6–13.0))	19	10.5	(6.0–15.0)	4	2.2	(0.0–4.4)	181
Manaus	41	12.2	(8.7–15.7)	66	19.7	(15.4–24.0)	17	5.1	(2.7–7.5)	335
Porto Alegre	22	11.6	(7.0–16.2)	23	12.2	(7.5–16.9)	4	2.1	(0.0–4.2)	189
Salvador	29	15.3	(10.1–20.5)	31	16.3	(11.0–21.6)	12	6.3	(2.8–9.8)	190
São Paulo	41	10.2	(7.2–13.2)	34	8.5	(5.8–11.2)	10	2.5	(1.0–4.0)	402
**Overall total**	149	11.5	(9.8–13.2)	173	13.3	(11.5–15.1)	47	3.6	(2.6–4.6)	1,297

95% CI: 95% confidence interval.

**Figure 1:**
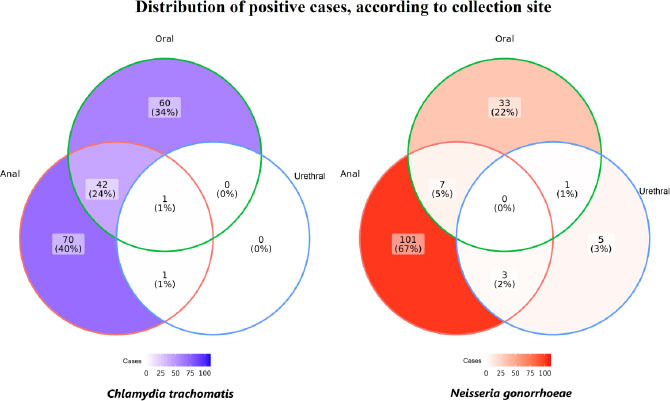
Venn diagram of positive cases of *Chlamydia trachomatis* and *Neisseria gonorrhoeae*, according to anatomical collection site, among transgender women and *travestis*, in five Brazilian capitals. TransOdara Study, 2019–2021.

The distribution of positive cases for CT and NG, according to the type of material (Figure 1), provides insight into the proportion of these infections occurring alone or concomitantly. In total, of the 111 participants with anorectal CT infection, 67% had only anorectal infections; 5% had anorectal and oropharyngeal infections; and 2%, anorectal and urethral infections. None showed infection in all biological materials. Of the 174 participants with detected NG, 40% had only anorectal infections, 34% had only oropharyngeal infections, 24% had both anorectal and oropharyngeal infections, while only 1% had anorectal and urethral infections, and 1% showed infections in all three sites: anorectal, oropharyngeal and urethral.

Of the 552 participants who underwent genital examination, only 1.3% had discharge, of which five were anal and two were urethral discharges.

Considering the total sample, the prevalence of anorectal infections by CT — 8.5% (95%CI 7.0–10.0) and by NG — 8.7% (95%CI 7.2–10.2), tended to be more prevalent than oropharyngeal infections — 3.2% (95%CI 2.2–4.2) and 7.9% (95%CI 6.4–9.4), respectively — and urethral ones were less prevalent — 0.7% (95%CI 0.2–1.2) and 0.2% (95%CI 0.0–0.4), but without a statistically significant difference. When comparing cities, for CT, anorectal infection rates varied from 9.5% (95%CI 5.3–13.7 — Salvador) to 6.1% (95%CI 2.6–9.6 — Campo Grande); oropharyngeal infection ranged from 5.8% (95%CI 2.5–9.1 — Salvador) to 1.2% (95%CI 0.1–2.3 — São Paulo); and urethral infections from 1.6% (95%CI 0.0–3.4 — Porto Alegre) to 0.5% (95%CI 0.0–1.5 — Salvador) — and São Paulo (95%CI 0. 0–1.2), but without statistical significance. For NG, anorectal infections ranged from 13.1% (95%CI 9.5–16.7 — Manaus) to 4.4% (95%CI 1.4–7.4 — Campo Grande), a statistically significant difference. The same occurred with oropharyngeal infections, which ranged from 12.2% (95%CI 8.7–15.7 — Manaus) to 5.2% (95%CI 3.0–7.4 — São Paulo). Urethral infections were least prevalent in Manaus: only 0.6% (95%CI 0.0–1.4) (Table 2).

**Table 2:** Prevalence estimates (n, %, and 95%CI) of chlamydia and gonorrhea among transgender women and *travestis*, according to anatomical collection site, in five Brazilian capitals. TransOdara Study, 2019–2021.

Characteristics	CT			NG		
	n	%	95%CI	n	%	95%CI
Campo Grande (n=181)						
Any site	16	8.8	(4.6–13.0)	19	10.5	(6.0–15.0)
Oral	5	2.8	(0.4–5.2)	13	7.2	(3.4–11.0)
Anal	11	6.1	(2.6–9.6)	8	4.4	(1.4–7.4)
Urethral	1	0.6	(0.0–1.72)	0	0.0	(0.0–0.0)
Manaus (n=335)						
Any site	41	12.2	(8.7–15.7)	66	19.7	(15.4–24.0)
Oral	14	4.2	(2.0–6.4)	41	12.2	(8.7–15.7)
Anal	28	8.4	(5.4–11.4)	44	13.1	(9.5–16.7)
Urethral	2	0.6	(0.0–1.4)	2	0.6	(0.0–1.4)
Porto Alegre (n=189)						
Any site	22	11.6	(7.0–16.2)	23	12.2	(7.5–16.9)
Oral	6	3.2	(0.7–5.7)	11	5.8	(2.4–9.2)
Anal	16	8.5	(4.5–12.5)	18	9.5	(5.3–13.7)
Urethral	3	1.6	(0.0–3.4)	0	0.0	(0.0–0.0)
Salvador (n=190)						
Any site	29	15.3	(10.1–20.5)	31	16.3	(11.0–21.6)
Oral	11	5.8	(2.5–9.1)	17	8.9	(4.8–13.0)
Anal	18	9.5	(5.3–13.7)	21	11.1	(6.6–15.6)
Urethral	1	0.5	(0.0–1.5)	0	0.0	(0.0–0.0)
São Paulo (n=402)						
Any site	41	10.2	(7.2–13.2)	34	8.5	(5.8–11.2)
Oral	5	1.2	(0.1–2.3)	21	5.2	(3.0–7.4)
Anal	37	9.2	(6.4–12.0)	22	5.5	(3.3–7.7)
Urethral	2	0.5	(0.0–1.2)	0	0.0	(0.0–0.0)
Overall total (n=1,297)						
Any site	149	11.5	(9.8–13.2)	173	13.3	(11.5–15.1)
Oral	41	3.2	(2.2–4.2)	103	7.9	(6.4–9.4)
Anal	110	8.5	(7.0–10.0)	113	8.7	(7.2–10.2)
Urethral	9	0.7	(0.2–1.2)	2	0.2	(0.0–0.4)

CT: *Chlamydia trachomatis*; NG: *Neisseria gonorrhoeae*; 95% CI: 95% confidence interval.

The prevalence estimates for each of the infections resulting from the univariate analyses and the multiple models, with both crude and adjusted ORs, according to sociodemographic and behavioral characteristics, are presented in Table 3 (CT), Table 4 (NG), and Table 5 (coinfection). It was observed that for all of them, both in the simple model and in the adjusted model, the chance of a greater occurrence was associated with younger people, seemingly decreasing in older age groups. Considering only the results of the adjusted model, they were associated with a greater chance of occurrence of CT: among individuals who engaged in sex work “only in the past” (OR=-1.73; 95%CI 1.02–2.95), “currently, part-time” (OR=2.75; 95%CI 1.60–4.75) and “currently, full-time” (OR=2.13; 95%CI 1.23–3.69), and those who reported having used injectable drugs in their lifetime (OR=3.54; 95%CI 1.49–8.40) (Table 3). For NG, the three categories of sexual orientation were associated — “heterosexual,” “bisexual,” and “homosexual” —, of which the last one had a higher chance of association (OR=5.49; 95%CI 1.89–15.97), and those who reported using injectable drugs in their lifetime (OR=1.91; 95%CI 1.28–2.84) (Table 4). For co-infection, those with the greatest chance of this condition occurring were those who performed sex work “currently, part-time” (OR=2.36; 95%CI 1.01–5.51), and those who reported having used drugs in the last 12 months (OR=2.34; 95% CI 1.10–5.00) (Table 5).

**Table 3:** Sociodemographic and behavioral characteristics (n and %, OR, adjusted OR, and 95% CI) of transgender women and *travesti*, according to chlamydia detection, in five Brazilian capitals. TransOdara Study, 2019–2021.

Characteristics	Chlamydia				Total (n=1,297)
	No (n=1,148)	Yes (n=149)	OR (95%CI)	Adjusted OR (95%CI)	
	n (%)	n (%)			n (%)
Age range (years)					
37 or more	373 (32.5)	30 (20.1)	1 - - -		403 (31.1)
26–36	460 (40.1)	59 (39.6)	**1.59 (1.01–2.53)**	1.56 (0.97–2.49)	519 (40.0)
18–25	315 (27.4)	60 (40.3)	**2.37 (1.49–3.76)**	**2.62 (1.63–4.22)**	375 (28.9)
Marital status					
Married	172 (15.0)	14 (9.4)	1 - - -		186 (14.3)
In a relationship	160 (13.9)	16 (10.7)	1.23 (0.58–2.60)	1.31 (0.61–2.80)	176 (13.6)
Single	812 (70.7)	119 (79.9)	**1.80 (1.01–3.21)**	1.79 (0.99–3.23)	931 (71.8)
N/A	4 (0.3)	0 (0.0)	N/A		4 (0.3)
Sex work					
Never	316 (27.5)	24 (16.1)	1 - - -		340 (26.2)
Only in the past	363 (31.6)	44 (29.5)	1.60 (0.95–2.68)	**1.73 (1.02–2.95)**	407 (31.4)
Current, part time	227 (19.8)	42 (28.2)	**2.44 (1.43–4.14)**	**2.75 (1.60–4.75)**	269 (20.7)
Current (main activity)	238 (20.7)	38 (25.5)	**2.10 (1.23–3.60)**	**2.13 (1.23–3.69)**	276 (21.3)
N/A	4 (0.3)	1 (0.7)	N/A		5 (0.4)
Drug use (last 12 months)					
No	424 (36.9)	39 (26.2)	1 - - -		463 (35.7)
Yes	724 (63.1)	110 (73.8)	**1.69 (1.13–2.54)**		834 (64.3)
Injectable drug use (in life)					
No	1,122 (97.7)	140 (94.0)	1 - - -		1,262 (97.3)
Yes	20 (1.7)	8 (5.4)	**3.21 (1.39–7.41)**	**3.54 (1.49–8.40)**	28 (2.2)
N/A	6 (0.5)	1 (0.7)	N/A		7 (0.5)

OR: odds ratio; 95% CI: 95% confidence interval. Bold: significant association (95%CI exceeded 1).

**Table 4:** Sociodemographic and behavioral characteristics (n and %, OR, adjusted OR, and 95%CI) of transgender women and *travesti*, according to detection of gonorrhea, in five Brazilian capitals. TransOdara Study, 2019–2021.

Characteristics	No (n=1,124)	Yes (n=173)	OR (95%CI)	Adjusted OR (95%CI)	Total (n=1,297)
	n (%)	n (%)			n (%)
Age range (years)					
37 or more	378 (33.6)	25 (14.5)	1 - - -		403 (31.1)
26–36	447 (39.8)	72 (41.6)	**2.43 (1.51–3.91)**	**2.21 (1.36–3.58)**	519 (40.0)
18–25	299 (26.6)	76 (43.9)	**3.55 (2.19–5.74)**	**3.70 (2.30–6.04)**	375 (28.9)
Sexual orientation					
Pansexual	76 (6.8)	5 (2.9)	1 - - -		81 (6.2)
Heterosexual	879 (78.2)	137 (79.2)	2.25 (0.89–5.71)	**3.44 (1.35–8.82)**	1016 (78.3)
Homosexual, gay or lesbian	79 (7.0)	18 (10.4)	**3.41 (1.20–9.69)**	**5.49 (1.89–15.97)**	97 (7.5)
Bisexual	73 (6.5)	12 (6.9)	2.52 (0.84–7.53)	**3.21 (1.06–9.68)**	85 (6.6)
Other	8 (0.7)	0 (0.0)	0 (0.00–6.44)		8 (0.6)
N/A			N/A		10 (0.8)
Marital status					
Married	170 (15.1)	16 (9.2)	1 - - -		186 (14.3)
In a relationship	161 (14.3)	15 (8.7)	0.95 (0.45–1.98)		176 (13.6)
Single	789 (70.2)	142 (82.1)	**1.78 (1.03–3.08)**		931 (71.8)
N/A	4 (0.4)	0 (0.0)	N/A		4 (0.3)
Sex work					
Never	307 (27.3)	33 (19.1)	1 - - -		340 (26.2)
Only in the past	358 (31.9)	49 (28.3)	1.25 (0.78–2.00)		407 (31.4)
Current, part time	226 (20.1)	43 (24.9)	**1.97 (1.21–3.23)**		269 (20.7)
Current (as main activity)	228 (20.3)	48 (27.7)	**2.21 (1.36–3.58)**		276 (21.3)
N/A	5 (0.4)	0 (0.0)	N/A		5 (0.4)
Drug use (last 12 months)					
No	414 (36.8)	49 (28.3)	1 - - -		463 (35.7)
Yes	710 (63.2)	124 (71.7)	**1.93 (1.31–2.82)**		834 (64.3)
Injectable drug use (in life)					
No	1,094 (97.3)	168 (97.1)	1 - - -		1,262 (97.3)
Yes	24 (2.1)	4 (2.3)	1.17 (0.40–3.42)	**1.91 (1.28–2.84)**	28 (2.2)
N/A	6 (0.5)	1 (0.6)	N/A		7 (0.5)

OR: odds ratio; 95% CI: 95% confidence interval. Bold: significant association (95%CI exceeded 1).

**Table 5:** Sociodemographic and behavioral characteristics (n and %, OR, adjusted OR, and 95% CI) of transgender women and *travestis*, according to detection of co-infection (chlamydia and gonorrhea), in five Brazilian capitals. TransOdara Study, 2019–2021.

Characteristics	No (n=1,250)	Yes (n=47)	Total (n=1,297)	OR (95%CI)	Adjusted OR (95%CI)
	n (%)	n (%)	n (%)		
Age range (years)					
37 or more	398 (31.8)	5 (10.6)	403 (31.1)	1 - - -	
26–36	498 (39.8)	21 (44.7)	519 (40.0)	**3.33 (1.25–8.91)**	**2.94 (1.10–7.87)**
18–25	354 (28.3)	21 (44.7)	375 (28.9)	**4.53 (1.67–12.28)**	**4.10 (1.52–11.05)**
Sex work					
Never	331 (26.5)	9 (19.1)	340 (26.2)	1 - - -	
Only in the past	397 (31.8)	10 (21.3)	407 (31.4)	0.91 (0.37–2.26)	
Current, part time	254 (20.3)	15 (31.9)	269 (20.7)	**2.36 (1.01–5.51)**	
Current, as main activity	263 (21.0)	13 (27.7)	276 (21.3)	1.94 (0.81–4.62)	
N/A	5 (0.4)	0 (0.0)	5 (0.4)	N/A	
Drug use (last 12 months)					
No	453 (36.2)	10 (21.3)	463 (35.7)	1 - - -	
Yes	797 (63.8)	37 (78.7)	834 (64.3)	**2.62 (1.25–5.51)**	**2.34 (1.10–5.00)**

OR: odds ratio; 95% CI: 95% confidence interval. Bold: significant association (95%CI exceeded 1).

The variables associated with each of the infections analyzed in the univariate model, but which did not remain associated in the multiple model, were: CT — “marital status,” “drug use in the last 12 months,” and “lifetime use of injectable drugs”; NG — “marital status,” “sex work,” and “drug use in the last 12 months”; and co-infection — “sex work.”

## DISCUSSION

Considering the point prevalence estimates of CT and NG infections among transgender women and *travestis*, this study revealed variations between cities and anatomical collection sites, although without statistically significant differences. The highest values were for anorectal infections, followed by oropharyngeal and urethral infections. Regarding sociodemographic characteristics, age (being younger) was the only common factor associated with a greater occurrence of CT, NG, and co-infections. For CT, other associated variables were “sex work” and “injectable drug use in life;” for NG, “injectable drug use in life,” as well as “sexual orientation;” and for co-infection, “sex work,” in addition to “drug use in the last 12 months” — associated only with this condition.

Chan et al.^10^ observed, in a systematic review (1981 and 2015), in the USA, that the prevalence of extragenital CT and NG infections in MSM varied depending on the anatomical site of collection. In this study, the values observed for anorectal CT involving transgender women and *travestis* were similar (8.5% *versus* 8.9%), but that for oropharyngeal CT was double the prevalence observed in that review (3.2% *versus* 1.7%).

Callander et al.^20^, in a study carried out in Australia (2010 to 2017), compared the prevalence of CT and NG infections between transgender (1,260) and cis populations — gay and bisexual men (387,848) —, seen in 46 STI clinics, and found that transgender women were 1.6 times more likely to acquire them. In the present study, the prevalence of CT was found to be similar to that reported in the Australian study (11.5% *versus* 10.0%). However, for NG, the prevalence was higher than that observed in Australia (13.3% *versus* 8.6%).

The systematic review conducted by Van Gerwen et al.^11^ included 25 studies carried out in 11 countries, including Brazil, focusing on the transsexual population. Among transgender women, the review reported prevalence rates of CT ranging from 2.7 to 24.7% and NG from 2.1 to 19.1%, with great variability according to anatomical site. The global prevalences obtained in the present study fell within these reported variations: 11.5% (95%CI 6.0–17.0) and 13.3% (95%CI 8.0–18.0), respectively.

When comparing the results of the present study with one conducted in Mexico^17^, with 206 MSM and six transgender women, it was found that the prevalence of CT, according to anatomical site, for both populations investigated, were similar for oropharyngeal (3.2% *versus* 2.8%), lower for anorectal (8.5% *versus* 11.8%) and urethral (0.2% *versus* 7.5%), as well as for NG infections — oropharyngeal (7.9% *versus* 7.5%), anorectal (8.7% *versus* 12.3%), and urethral (0.7% *versus* 4.2%). The higher prevalence of urethral infections observed in the Mexican study may be attributed to the predominantly MSM composition of the sample. Furthe more, both studies highlighted that the majority of CT and NG infections were asymptomatic, consistent with observations in the existing literature^13^.

In Brazil, few studies have investigated the prevalence of CT and NG infections in the transgender population, making comparisons with the present study challenging. In relation to diverse populations, the results observed were greater than those obtained in parturient women^24^, in sexually active young women^16,25^, and in women with HIV^22^. A study conducted in six Brazilian cities involving 767 men attending STI clinics reported higher prevalence rates compared to those observed in this study: CT (13.1% *versus* 11.5%) and NG (18.4% *versus* 13.3%)^21^.

Research conducted in Salvador with 246 transgender women and *travestis* and MSM aged 15 to 19 years, from a PrEP1519 cohort study between 2019 and 2021, found prevalences of: 5.9% for CT infection (1.2% oropharyngeal, 2.4% anorectal, and 1.9% urethral); and 17.9% for NG infection (9.4% oropharyngeal, 7.8% anorectal, and 1.9% urethral)^19^. These prevalences were lower than those observed in the samples collected in Salvador in the present study — respectively, CT and NG: global (15.3 and 16.3%), oropharyngeal (5.8 and 8.9%), anorectal (9.5 and 11.1%), and urethral (0.5 and 0.0%).

A study conducted in Rio de Janeiro, with 391 MSM with and without HIV, also observed higher prevalences of CT than those in the present study — anorectal (10.4% *versus* 8.5%) and urethral (2.2% *versus* 0 .7%). Conversely, lower rates were noted for anorectal NG (2.5% *versus* 8.7%)^18^.

The prevalence of co-infection in this study was higher than that found by Piazetta et al. for young women in Southern Brazil^25^ (3.6% *versus* 0.9%) and by Oliveira et al. for MSM and transgender adolescents in Salvador (3.6% *versus* 0.5%)^19^.

Variations in prevalence estimates of these infections across different studies can be explained by the involvement of populations with diverse sexual practices. International studies include more users of STI clinics, likely comprising individuals with more complaints and symptoms, leading to a higher prevalence of CT and NG compared to the general population. In this sense, the present study stands out for having achieved a robust sample, obtained through RDS in five capitals, representative of Brazilian macro-regions. Despite the limitations of this recruitment technique, the study’s estimates are likely closer to real-world figures.

In the present study, although no statistically significant difference was observed between cities, the values of point prevalence estimates show great variation, probably due to difficulties in access and the varying levels of social inequalities inherent in Brazilian macro-regions. The highest point prevalences observed for CT infections and co-infections were in Salvador, while for NG infection, in Manaus.

Among the factors associated with these infections, as observed in the present study, young age, sex work, and the type of sexual practice, with the exception of drug use, also appear to be associated in other studies^2,3,12,17,21^.

In a systematic review involving STI studies carried out in several countries, it was found that the prevalence of CT and NG in transgender women showed great variability. The highest prevalences were observed in young people under 25 years of age, sexual and gender minorities, transgender women who have sex with men, black people and Latinos^11^. The similar behavior of the associated variables may indicate that the differences are associated with lower statistical power, given the smaller number of positive cases for both infections simultaneously.

The RDS method has been extensively employed in studies with populations that are challenging to recruit using other sampling methods. However, the results need to be interpreted with caution as they may only be representative of the social networks eventually captured by the study, whose characteristics are generally unknown. In the absence of better strategies, RDS continues to be a method of choice for studies involving populations of transgender women and *travestis* in various contexts^31^.

The present study contributed to updating the epidemiology of STIs among transgender women and *travestis* and confirms that they occur with greater magnitude compared to the general population. As the majority of cases are asymptomatic, controlling these infections remains a challenge for public health, especially among younger people, those engaged in sex work, and those who use drugs.

Transgender women and *travestis* constitute a vulnerable population, facing barriers that hinder their access to health services, highlighting the inadequacy of existing legislation to address this issue^32^. The heightened vulnerability of this population group may be associated with the multiplicity of sexual partners and sexual violence, culminating in an increased risk of STIs^33^. The characteristics observed among transgender women and *travestis* in this study further underscore their vulnerability, which confirms the importance of prioritizing them for prevention and treatment actions targeting the investigated STIs.

These findings also confirm the importance of incorporating a comprehensive sexual history assessment into routine care, especially for populations at higher epidemiological risk, in addition to making screening available to detect these infections as a public health policy. This recommendation is already included in several clinical protocols for STIs, including the Brazilian one^15^.
