# Supplementary material for: Prevalence of chlamydia and gonorreheae among transgender women and travestis in five Brazilian capitals, 2019–2021
Source: Rev Bras Epidemiol. 2024 Aug 19;27(Suppl 1):e240006.supl.1. doi: 10.1590/1980-549720240006.supl.1 (PMC11338534; doi:10.1590/1980-549720240006.supl.1)
Supplement: Translation

# Translation: Prevalências de clamídia e gonorreia entre mulheres trans e travestis de cinco capitais brasileiras, 2019–2021

**DOI:** 10.1590/1980-549720240006.supl.1.2

**Authors:** Katia Cristina Bassichetto, Sandro Sperandei, Daniel Jason McCartney, Carla Gianna Luppi, Roberto José Carvalho da Silva, Sandra Araújo, Laio Magno, Maria Luíza Bazzo, Gwenda Hughes, Philippe Mayaud, Inês Dourado, Maria Amélia de Sousa Mascena Veras

**Affiliations:** I Santa Casa de São Paulo, Faculdade de Ciências Médicas – São Paulo (SP), Brasil.; II Western Sydney University, Translational Health Research Institute – Sydney, Australia.; III London School of Hygiene & Tropical Medicine, Faculty of Infectious & Tropical Diseases, Department of Clinical Research – London, United Kingdom.; IV Secretaria de Estado da Saúde de São Paulo, Centro de Referência e Treinamento em DST/Aids – São Paulo (SP), Brasil.; V Universidade do Estado da Bahia, Departamento de Ciências da Vida – Salvador (BA), Brasil.; VI Universidade Federal de Santa Catarina, Programa de Pós-graduação em Farmácia/Centro de Ciências da Saúde, Laboratório de Biologia Molecular, Microbiologia e Sorologias – Florianópolis (SC), Brasil.; VII Universidade Federal da Bahia, Instituto de Saúde Coletiva – Salvador (BA), Brasil.

**Keywords:** Epidemiologia, Infecções sexualmente transmissíveis, Mulher transexual, Travesti, Chlamydia, Gonorreia

## Abstract

**Objetivo:**

Estimar as prevalências e os fatores associados à detecção de *Chlamydia trachomatis* (CT) e *Neisseria gonorrhoeae* (NG) em mulheres trans e travestis em cinco capitais brasileiras.

**Métodos:**

Os dados vieram de um estudo transversal, realizado entre 2019 e 2021, com pessoas recrutadas por RDS (*respondent driven sampling*) em São Paulo, Campo Grande, Manaus, Porto Alegre e Salvador. Analisou-se a detecção de CT e NG, em três sítios de coleta (anorretal, orofaríngeo e uretral). Para identificação dos fatores associados empregaram-se modelos logísticos com efeitos mistos.

**Resultados:**

Forneceram material biológico para detecção dessas infecções 1.297 participantes recrutadas. As prevalências de CT, NG e coinfecção foram, respectivamente, 11,5, 13,3 e 3,6%. Foram independentemente associados à detecção para CT: trabalho sexual no passado (*odds ratio* — OR=1,73; intervalos de confiança para 95% — IC95% 1,02–2,95), no momento atual (OR=2,13; IC95% 1,23–3,69) e como atividade parcial (OR=2,75; IC95% 1,60–4,75) e uso de drogas injetáveis na vida (OR=3,54; IC95% 1,49–8,40). Para NG: uso de drogas injetáveis na vida (OR=1,91; IC95% 1,28–2,84) e orientação sexual – heterossexuais (OR=3,44; IC95% 1,35–8,82), homossexuais (OR=5,49; IC95% 1,89–15,97) e bissexuais (OR=3,21; IC95% 1,06–9,68). Para coinfecção: uso de drogas nos últimos 12 meses (OR=2,34; IC95% 1,10–5,00). Ser mais jovem foi associada a todos os desfechos investigados.

**Conclusão:**

As prevalências estimadas de CT, NG e de coinfecção foram desproporcionalmente mais elevadas entre as mulheres trans e travestis se comparadas à população geral, especialmente entre as mais jovens, que exerciam trabalho sexual e faziam uso de drogas.

## INTRODUÇÃO

A Organização Mundial de Saúde (OMS) estimou aproximadamente 374 milhões de novas infecções sexualmente transmissíveis (IST) em 2020, estando entre as mais prevalentes as por *Chlamydia trachomatis* (CT) e *Neisseria gonorrhoeae* (NG), com, respectivamente, 128,5 e 82,4 milhões de casos, sendo 29,8 e 13,8 milhões nas Américas. Constituem um problema de saúde pública, gerando elevados custos^1^. A coinfecção apresenta grande variação entre países e grupos populacionais. Há poucos estudos na população trans, conhecimento fundamental para orientar intervenções e aprimorar políticas para essa população^2-5^.

A prevenção e o controle da CT e da NG estão previstos nas estratégias globais para a saúde e incluem o compromisso de redução da incidência de NG em 90% até 2030, com foco em populações mais vulnerabilizadas para IST^6^, e o fortalecimento do Programa de Resistência Antimicrobiana para Gonorreia (GASP)^7-9^.

As prevalências dessas infecções têm sido investigadas em diversos contextos, sendo elevadas entre homens que fazem sexo com homens (HSH) e mulheres trans, e apresentam grande variabilidade, a depender do sítio anatômico^10,11^. Mais da metade das infecções extragenitais é assintomática ou oligossintomática, contribuindo para manter a cadeia de transmissão^12^.

Diversas instituições internacionais de saúde pública e sexual têm recomendado a disponibilização do rastreamento de CT e NG para as populações de maior risco, incluindo a transexual^6^.

No Brasil, essas estratégias continuam um desafio, especialmente pela dificuldade da ampliação da detecção do agente etiológico no Sistema Único de Saúde (SUS), ainda restrita às unidades sentinelas, porém há perspectivas de expansão^7,13,14^.

O Protocolo Clínico e Diretrizes Terapêuticas (PCDT) para Atenção Integral às Pessoas com IST, elaborado pelo Ministério da Saúde (MS)^15^, recomenda o rastreamento semestral, com coleta de swab anal para detecção de CT e NG, para as pessoas com prática anal receptiva, sem proteção de barreira. Para viabilizar o diagnóstico e tratamento adequados de assintomáticos(as) e parceiros(as), o MS tem ofertado testes para detecção desses patógenos, buscando atender às prioridades no cuidado integral às pessoas com IST^15^. Entretanto, o rastreamento ainda não está completamente implantado na rotina de atenção e muitas infecções extragenitais não têm sido diagnosticadas, podendo levar a complicações, como dor crônica e proctite^16^, e aumentando o risco de transmissão de HIV^15^.

Alguns estudos investigaram a prevalência de CT e NG em populações: HSH^2,5,17-19^, usuários de clínicas de IST^3,4,20,21^, pessoas com HIV^22,23^, parturientes^24^, mulheres em serviços de reprodução humana^16^ e mulheres jovens sexualmente ativas^25^. Quando envolvem a população trans, esta se encontra em amostras reduzidas ou restritas a serviços especializados^2,10,12,17,19,20^. É importante realizar estimativas dessas infecções em mulheres trans e travestis, recrutadas por pares e não somente vinculadas a serviços especializados.

O objetivo deste estudo foi estimar as prevalências e os fatores associados às infecções por CT, NG e coinfecção, segundo sítio anatômico (anal, oral e uretral) e presença de sinais e sintomas, em mulheres trans e travestis, em cinco capitais brasileiras.

## MÉTODOS

### Desenho

Foram utilizados dados do TransOdara, estudo transversal com abordagem quantitativa e qualitativa, realizado entre 2019 e 2021, em cinco capitais brasileiras (São Paulo, Campo Grande, Manaus, Porto Alegre e Salvador), para estimar a prevalência de oito IST, incluindo CT e NG. Procurou ainda demonstrar a viabilidade da estratégia *point-of-care* em fornecer, quando possível, recursos para prevenção e tratamento disponíveis no SUS (profilaxias pré- e pós-exposição ao HIV e vacinas contra hepatite B e HPV) e/ou tratamentos para IST em geral, no mesmo dia da participação na pesquisa ou por meio de encaminhamentos mediados pelos(as) pesquisadores(as)^26^.

A coleta de dados foi realizada nos locais designados para o desenvolvimento da pesquisa, e o fluxo previa desde confirmação de elegibilidade, oferta de testes laboratoriais, entrevista e aplicação de questionário sociocomportamental, pré- e pós-consulta de enfermagem, consulta médica, incluindo exame físico, independentemente da presença de sinais e sintomas e encaminhamentos quando indicados. Os instrumentos utilizados foram os Formulários de Pré-elegibilidade, de Aceitabilidade de Coleta Pré-Consulta e de Procedimentos Pré-Consulta, de Avaliação Clínica e Seguimento, de Aceitabilidade de Coleta e de Procedimentos Pós-Consulta e de Avaliação Laboratorial, além do questionário sociocomportamental.

### Amostra

A técnica de amostragem utilizada foi *respondent driven sampling* (RDS), apropriada para recrutar populações de difícil acesso^27^, sendo incluídas 1.317 participantes. O detalhamento das estratégias de recrutamento, dos critérios de elegibilidade, do cálculo da amostra e do fluxo da pesquisa está no artigo metodológico, neste suplemento^26^.

### Prevalências de clamídia e gonorreia e variáveis selecionadas

Foram realizadas coletas (por profissional de saúde ou por autocoleta) de amostras de urina, swab retal e orofaríngeo com o *Multi-collect kit*, a depender da opção da participante. As que optaram por autocoleta receberam orientação padronizada, incluindo folhetos ilustrativos, e a realizaram no próprio local da pesquisa.

A variável dependente foi o resultado detectável para CT ou NG, em pelo menos um dos sítios de coleta e para coinfecção CT/NG, segundo cidades de realização da pesquisa e presença de sinais e sintomas (somente corrimento uretral, somente corrimento anal ou ambos).

As amostras coletadas em São Paulo (Centro de Referência e Tratamento em IST/Aids de São Paulo) eram levadas diariamente para o laboratório que funcionava na mesma unidade na qual a pesquisa foi realizada. As coletadas nas demais capitais foram enviadas por via aérea para São Paulo, acondicionadas em caixas de transporte com gelo seco para análise nesse mesmo laboratório. Imediatamente após o recebimento, eram conservadas no *Multi*-*collect kit* e armazenadas em freezer a -20°C, até que se realizasse o processamento no equipamento M2000 SP/RT da Abbott. A estabilidade prevista das amostras após coleta era de 90 dias.

O método de detecção para CT e NG foi Reação em Cadeia da Polimerase (PCR), e os critérios para estabelecer a validade do procedimento do ensaio e para determinação qualitativa do plasmídeo de DNA de CT e do DNA genômico de NG em amostras de swabs foram *Abbott Real Time CT/NG Controls*.

As variáveis independentes analisadas para caracterização socioeconômica e demográfica foram: idade (18–25; 26–36; 37 anos ou mais); raça/cor (branca; preta/parda; outra); religião (sem religião; afro-brasileira; evangélica; católica); orientação sexual (pansexual; heterossexual; homossexual, gay ou lésbica); situação conjugal (casada; em um relacionamento; solteira); escolaridade (ensino fundamental; ensino médio e universitário); situação de moradia (própria; alugada; com amigos/família; instável); trabalho sexual (nunca; apenas no passado; atual, parcialmente; atual como atividade principal); e as demais categorizadas em “sim/não”: uso de drogas nos últimos 12 meses e uso de drogas injetáveis na vida, histórico de encarceramento, histórico de agressão, consentimento na primeira relação sexual, uso de preservativo na primeira relação sexual, com último parceiro fixo e com último parceiro casual.

#### Análise estatística

Foi realizada análise descritiva para as variáveis explicativas, apresentadas como frequências absolutas e relativas, construídos modelos logísticos univariados com efeitos mistos para as cidades de coleta e selecionadas as variáveis com p-valor menor ou igual a 0,3 para construção dos modelos multivariados finais^28^. Para tanto, utilizou-se o pacote StatisticalModels, a estratégia *backward stepwise* e o critério da máxima verossimilhança. Não foram utilizados os pesos RDS por não apresentarem vantagens e por poderem dar origem a resultados enviesados^29^. Os resultados dos modelos logísticos foram apresentados em razão de chances (*odds ratio* – OR) e intervalos de confiança para 95% (IC95%). Todas as análises foram realizadas em R, versão 4.3.1^30^.

#### Aspectos éticos

O projeto foi aprovado pelo Comitê de Ética em Pesquisa (CEP) da Santa Casa de Misericórdia de São Paulo (CAAE 05585518.7.0000.5479 - Nº parecer: 3.126.815 - 30/01/2019), assim como pelas demais instituições participantes. O consentimento informado foi obtido de todas as participantes incluídas^26^.

## RESULTADOS

Neste estudo, forneceram os materiais biológicos (urina, swab orofaringe e/ou anal) para detecção de CT e/ou NG, 98,5% das participantes recrutadas no TransOdara (1.297/1.317) — Campo Grande (n=181), Manaus (n=335), Porto Alegre (n=189), Salvador (n=190) e São Paulo (n=402). A média de idade dessas participantes foi de 32,5 anos (desvio-padrão – DP=9,88). A maioria (70%) se autorreferiu com cor da pele preta/parda; 36,5% sem religião; 78,3% heterossexual; 71,8% solteira; e 70% cursando ou com o ensino médio completo; cerca de 85% das participantes viviam com baixa renda — 43,8% recebiam menos de um salário mínimo; 36,2% viviam em moradia alugada e 21,3% exerciam o trabalho sexual como ocupação principal. O uso de drogas nos últimos 12 meses e o uso de drogas injetáveis em algum momento da vida foram referidos, respectivamente, por 64,3 e 2,2% delas; 22,7% já haviam sido presas e 50,3% já haviam sofrido agressão sexual alguma vez na vida; 14,2% referiram que a primeira relação sexual foi forçada; e nos últimos 6 meses, 31 e 16,3% não usaram preservativo com o(a) último(a) parceiro(a) fixo(a) ou com o(a) parceiro(a) comercial, respectivamente.

As prevalências dessas infecções, segundo as cidades, constam da Tabela 1. Das 1.297 participantes, 11,5% (IC95% 9,8–13,2) apresentaram detecção para CT, 13,3% (IC95% 11,5–15,1) para NG e 3,6% (IC95% 2,6–4,6) para coinfecção. Independentemente do tipo de material, as prevalências para CT variaram entre 15,3% (IC95% 10,1–20,5) (Salvador) e 8,8% (IC95% 4,6–13,0) (Campo Grande); para NG, entre 19,7% (IC95% 15,4–24,0) (Manaus) e 8,5% (IC95% 5,8–11,2) (São Paulo); e para coinfecção, entre 6,3% (IC95% 2,8–9,8) (Salvador) e 2,1% (IC95% 0,0–4,2) (Porto Alegre), não sendo verificada diferença estatisticamente significante segundo elas.

**Tabela 1:** Estimativas de prevalência (n, % e IC95%) de clamídia, gonorreia e coinfecção, entre mulheres trans e travestis, em cinco capitais brasileiras. Estudo TransOdara, 2019–2021.

Cidades	Clamídia			Gonorreia			Coinfecção			
	n	%	IC95%	n	%	IC95%	n	%	IC95%	n
Campo Grande	16	8,8	(4,6–13,0))	19	10,5	(6,0–15,0)	4	2,2	(0,0–4,4)	181
Manaus	41	12,2	(8,7–15,7)	66	19,7	(15,4–24,0)	17	5,1	(2,7–7,5)	335
Porto Alegre	22	11,6	(7,0–16,2)	23	12,2	(7,5–16,9)	4	2,1	(0,0–4,2)	189
Salvador	29	15,3	(10,1–20,5)	31	16,3	(11,0–21,6)	12	6,3	(2,8–9,8)	190
São Paulo	41	10,2	(7,2–13,2)	34	8,5	(5,8–11,2)	10	2,5	(1,0–4,0)	402
**Total geral**	149	11,5	(9,8–13,2)	173	13,3	(11,5–15,1)	47	3,6	(2,6–4,6)	1.297

IC95%: intervalo de confiança para 95%.

A distribuição de casos positivos para CT e NG, segundo tipo de material (Figura 1), permite verificar em que proporção essas infecções se apresentam isoladamente ou concomitantemente. No total, das 111 participantes com infecção anorretal para CT, 67% apresentaram somente anorretais; 5%, anorretais e orofaríngeas; e 2%, anorretais e uretrais. Nenhuma apresentou infecção em todos os materiais biológicos. Das 174 que tiveram detecção de NG, 40% foram só anorretais, 34% só orofaríngeas, 24% anorretais e orofaríngeas, apenas 1% anorretal e uretral, e 1% anal, oral e uretral.

**Figura 1:** Diagrama de Venn de casos positivos de *Chlamydia trachomatis* e *Neisseria gonorrhoeae*, segundo sítio anatômico de coleta, entre mulheres trans e travestis, em cinco capitais brasileiras. Estudo TransOdara, 2019–2021.

Das 552 participantes que permitiram exame genital, apenas 1,3% apresentaram corrimento, das quais cinco era corrimento anal e duas corrimento uretral.

Considerando o total da amostra, as prevalências das infecções anorretais por CT — 8,5% (IC95% 7,0–10,0) e por NG — 8,7% (IC95% 7,2–10,2), tenderam a ser mais prevalentes do que as orofaríngeas — 3,2% (IC95% 2,2–4,2) e 7,9% (IC95% 6,4–9,4), respectivamente —, e as uretrais foram menos prevalentes — 0,7% (IC95% 0,2–1,2) e 0,2% (IC95% 0,0–0,4), porém sem diferença estatisticamente significante. Na comparação entre as cidades, para CT, as anorretais variaram de 9,5% (IC95% 5,3–13,7 — Salvador) a 6,1% (IC95% 2,6–9,6 — Campo Grande); as orofaríngeas, de 5,8% (IC95% 2,5–9,1 — Salvador) a 1,2% (IC95% 0,1–2,3 — São Paulo); e as uretrais, de 1,6% (IC95% 0,0–3,4 — Porto Alegre) a 0,5% (IC95% 0,0–1,5 — Salvador) — e São Paulo (IC95% 0,0–1,2), porém sem significância estatística. Para NG, as infecções dos sítios anorretais variaram de 13,1% (IC95% 9,5–16,7 — Manaus) a 4,4% (IC95% 1,4–7,4 — Campo Grande), diferença estatisticamente significante. O mesmo ocorreu com as orofaríngeas, que variaram de 12,2% (IC95% 8,7–15,7 — Manaus) a 5,2% (IC95% 3,0–7,4 — São Paulo). As menos prevalentes foram as uretrais, em Manaus: apenas 0,6% (IC95% 0,0–1,4) (Tabela 2).

**Tabela 2:** Estimativas de prevalência (n, % e IC95%) de clamídia e gonorreia entre mulheres trans e travestis, segundo sítio anatômico de coleta, em cinco capitais brasileiras. Estudo TransOdara, 2019–2021.

Variáveis	CT			NG		
	n	%	IC95%	n	%s	IC95%
Campo Grande (n=181)						
Qualquer sítio	16	8,8	(4,6–13,0)	19	10,5	(6,0–15,0)
Oral	5	2,8	(0,4–5,2)	13	7,2	(3,4–11,0)
Anal	11	6,1	(2,6–9,6)	8	4,4	(1,4–7,4)
Uretral	1	0,6	(0,0–1,72)	0	0,0	(0,0–0,0)
Manaus (n=335)						
Qualquer sítio	41	12,2	(8,7–15,7)	66	19,7	(15,4–24,0)
Oral	14	4,2	(2,0–6,4)	41	12,2	(8,7–15,7)
Anal	28	8,4	(5,4–11,4)	44	13,1	(9,5–16,7)
Uretral	2	0,6	(0,0–1,4)	2	0,6	(0,0–1,4)
Porto Alegre (n=189)						
Qualquer sítio	22	11,6	(7,0–16,2)	23	12,2	(7,5–16,9)
Oral	6	3,2	(0,7–5,7)	11	5,8	(2,4–9,2)
Anal	16	8,5	(4,5–12,5)	18	9,5	(5,3–13,7)
Uretral	3	1,6	(0,0–3,4)	0	0,0	(0,0–0,0)
Salvador (n=190)						
Qualquer sítio	29	15,3	(10,1–20,5)	31	16,3	(11,0–21,6)
Oral	11	5,8	(2,5–9,1)	17	8,9	(4,8–13,0)
Anal	18	9,5	(5,3–13,7)	21	11,1	(6,6–15,6)
Uretral	1	0,5	(0,0–1,5)	0	0,0	(0,0–0,0)
São Paulo (n=402)						
Qualquer sítio	41	10,2	(7,2–13,2)	34	8,5	(5,8–11,2)
Oral	5	1,2	(0,1–2,3)	21	5,2	(3,0–7,4)
Anal	37	9,2	(6,4–12,0)	22	5,5	(3,3–7,7)
Uretral	2	0,5	(0,0–1,2)	0	0,0	(0,0–0,0)
Total geral (n=1.297)						
Qualquer sítio	149	11,5	(9,8–13,2)	173	13,3	(11,5–15,1)
Oral	41	3,2	(2,2–4,2)	103	7,9	(6,4–9,4)
Anal	110	8,5	(7,0–10,0)	113	8,7	(7,2–10,2)
Uretral	9	0,7	(0,2–1,2)	2	0,2	(0,0–0,4)

CT: *Chlamydia trachomatis*; NG: *Neisseria gonorrhoeae*; IC95%: intervalos de confiança para 95%.

As estimativas de prevalências para cada uma das infecções, resultantes das análises univariada e do modelo múltiplo, com OR brutos e ajustados, segundo características sociodemográficas e comportamentais, estão apresentadas na Tabela 3 (CT), na Tabela 4 (NG) e na Tabela 5 (coinfecção). Observou-se que para todas elas, tanto no modelo simples quanto no modelo ajustado, a chance de maior ocorrência esteve associada às mais jovens, parecendo diminuir nas faixas etárias mais velhas. Considerando apenas os resultados do modelo ajustado, mostraram-se associadas à maior chance de ocorrência de CT: as que exerciam trabalho sexual “apenas no passado” (OR=-1,73; IC95% 1,02–2,95), “no momento atual, parcialmente” (OR=2,75; IC95% 1,60–4,75) e “no momento atual, em período integral” (OR=2,13; IC95% 1,23–3,69), e as que referiram uso de drogas injetáveis na vida (OR=3,54; IC95% 1,49–8,40) (Tabela 3). Para NG, mostraram-se associadas as três categorias de orientação sexual — “heterossexuais”, “bissexuais” e “homossexuais” —, das quais a última apresentou pontualmente maior chance de associação (OR=5,49; IC95% 1,89–15,97), e as que referiram uso de drogas injetáveis na vida (OR=1,91; IC95% 1,28–2,84) (Tabela 4). Para coinfecção, as que apresentaram maior chance de ocorrência dessa condição foram as que exerciam trabalho sexual “no momento atual, em período parcial” (OR=2,36; IC95% 1,01–5,51), e as que referiram uso de drogas nos últimos 12 meses (OR=2,34; IC95% 1,10–5,00) (Tabela 5).

**Tabela 3:** Características sociodemográficas e comportamentais (n e %, OR, OR ajustado e IC95%) de mulheres trans e travestis, segundo detecção de clamídia, em cinco capitais brasileiras. Estudo TransOdara, 2019–2021.

Variáveis	Clamídia				Total (n=1.297)
	Não (n=1.148)	Sim (n=149)	OR (IC95%)	OR ajustado (IC95%)	
	n (%)	n (%)			n (%)
Faixa etária (anos)					
37 ou mais	373 (32,5)	30 (20,1)	1 - - -		403 (31,1)
26–36	460 (40,1)	59 (39,6)	**1,59 (1,01–2,53)**	1,56 (0,97–2,49)	519 (40,0)
18–25	315 (27,4)	60 (40,3)	**2,37 (1,49–3,76)**	**2,62 (1,63–4,22)**	375 (28,9)
Situação conjugal					
Casada	172 (15,0)	14 (9,4)	1 - - -		186 (14,3)
Em um relacionamento	160 (13,9)	16 (10,7)	1,23 (0,58–2,60)	1,31 (0,61–2,80)	176 (13,6)
Solteira	812 (70,7)	119 (79,9)	**1,80 (1,01–3,21)**	1,79 (0,99–3,23)	931 (71,8)
N/A	4 (0,3)	0 (0,0)	N/A		4 (0,3)
Trabalho sexual					
Nunca	316 (27,5)	24 (16,1)	1 - - -		340 (26,2)
Apenas no passado	363 (31,6)	44 (29,5)	1,60 (0,95–2,68)	**1,73 (1,02–2,95)**	407 (31,4)
Atual, parcialmente	227 (19,8)	42 (28,2)	**2,44 (1,43–4,14)**	**2,75 (1,60–4,75)**	269 (20,7)
Atual (atividade principal)	238 (20,7)	38 (25,5)	**2,10 (1,23–3,60)**	**2,13 (1,23–3,69)**	276 (21,3)
N/A	4 (0,3)	1 (0,7)	N/A		5 (0,4)
Uso de drogas (últimos 12 meses)					
Não	424 (36,9)	39 (26,2)	1 - - -		463 (35,7)
Sim	724 (63,1)	110 (73,8)	**1,69 (1,13–2,54)**		834 (64,3)
Uso de drogas injetáveis (na vida)					
Não	1.122 (97,7)	140 (94,0)	1 - - -		1.262 (97,3)
Sim	20 (1,7)	8 (5,4)	**3,21 (1,39–7,41)**	**3,54 (1,49–8,40)**	28 (2,2)
N/A	6 (0,5)	1 (0,7)	N/A		7 (0,5)

OR: *odds ratio*; IC95%: intervalos de confiança para 95%. Negrito: associação significativa (o IC95% passou de 1).

**Tabela 4:** Características sociodemográficas e comportamentais (n e %, OR, OR ajustado e IC95%) de mulheres trans e travestis, segundo detecção de gonorreia, em cinco capitais brasileiras. Estudo TransOdara, 2019–2021.

Variáveis	Não (n=1.124)	Sim (n=173)	OR (IC95%)	OR ajustado (IC95%)	Total (n=1.297)
	n (%)	n (%)			n (%)
Faixa etária (anos)					
37 ou mais	378 (33,6)	25 (14,5)	1 - - -		403 (31,1)
26–36	447 (39,8)	72 (41,6)	**2,43 (1,51–3,91)**	**2,21 (1,36–3,58)**	519 (40,0)
18–25	299 (26,6)	76 (43,9)	**3,55 (2,19–5,74)**	**3,70 (2,30–6,04)**	375 (28,9)
Orientação sexual					
Pansexual	76 (6,8)	5 (2,9)	1 - - -		81 (6,2)
Heterossexual	879 (78,2)	137 (79,2)	2,25 (0,89–5,71)	**3,44 (1,35–8,82)**	1016 (78,3)
Homossexual, gay ou lésbica	79 (7,0)	18 (10,4)	**3,41 (1,20–9,69)**	**5,49 (1,89–15,97)**	97 (7,5)
Bissexual	73 (6,5)	12 (6,9)	2,52 (0,84–7,53)	**3,21 (1,06–9,68)**	85 (6,6)
Outra	8 (0,7)	0 (0,0)	0 (0,00–6,44)		8 (0,6)
N/A			N/A		10 (0,8)
Situação conjugal					
Casada	170 (15,1)	16 (9,2)	1 - - -		186 (14,3)
Em um relacionamento	161 (14,3)	15 (8,7)	0,95 (0,45–1,98)		176 (13,6)
Solteira	789 (70,2)	142 (82,1)	**1,78 (1,03–3,08)**		931 (71,8)
N/A	4 (0,4)	0 (0,0)	N/A		4 (0,3)
Trabalho sexual					
Nunca	307 (27,3)	33 (19,1)	1 - - -		340 (26,2)
Apenas no passado	358 (31,9)	49 (28,3)	1,25 (0,78–2,00)		407 (31,4)
Atual, parcialmente	226 (20,1)	43 (24,9)	**1,97 (1,21–3,23)**		269 (20,7)
Atual (atividade principal)	228 (20,3)	48 (27,7)	**2,21 (1,36–3,58)**		276 (21,3)
N/A	5 (0,4)	0 (0,0)	N/A		5 (0,4)
Uso de drogas (últimos 12 meses)					
Não	414 (36,8)	49 (28,3)	1 - - -		463 (35,7)
Sim	710 (63,2)	124 (71,7)	**1,93 (1,31–2,82)**		834 (64,3)
Uso de drogas injetáveis (na vida)					
Não	1.094 (97,3)	168 (97,1)	1 - - -		1.262 (97,3)
Sim	24 (2,1)	4 (2,3)	1,17 (0,40–3,42)	**1,91 (1,28–2,84)**	28 (2,2)
N/A	6 (0,5)	1 (0,6)	N/A		7 (0,5)

OR: *odds ratio*; IC95%: intervalos de confiança para 95%. Negrito: associação significativa (o IC95% passou de 1).

**Tabela 5:** Características sociodemográficas e comportamentais (n e %, OR, OR ajustado e IC95%) de mulheres trans e travestis, segundo detecção de coinfecção (clamídia e gonorreia), em cinco capitais brasileiras. Estudo TransOdara, 2019–2021.

Variáveis	Não (n=1.250)	Sim (n=47)	Total (n=1.297)	OR (IC95%)	OR ajustado (IC95%)
	n (%)	n (%)	n (%)		
Faixa etária (anos)					
37 ou mais	398 (31,8)	5 (10,6)	403 (31,1)	1 - - -	
26–36	498 (39,8)	21 (44,7)	519 (40,0)	**3,33 (1,25–8,91)**	**2,94 (1,10–7,87)**
18–25	354 (28,3)	21 (44,7)	375 (28,9)	**4,53 (1,67–12,28)**	**4,10 (1,52–11,05)**
Trabalho sexual					
Nunca	331 (26,5)	9 (19,1)	340 (26,2)	1 - - -	
Apenas no passado	397 (31,8)	10 (21,3)	407 (31,4)	0,91 (0,37–2,26)	
Atual, parcialmente	254 (20,3)	15 (31,9)	269 (20,7)	**2,36 (1,01–5,51)**	
Atual, como atividade principal	263 (21,0)	13 (27,7)	276 (21,3)	1,94 (0,81–4,62)	
N/A	5 (0,4)	0 (0,0)	5 (0,4)	N/A	
Uso de drogas (últimos 12 meses)					
Não	453 (36,2)	10 (21,3)	463 (35,7)	1 - - -	
Sim	797 (63,8)	37 (78,7)	834 (64,3)	**2,62 (1,25–5,51)**	**2,34 (1,10–5,00)**

OR: *odds ratio*; IC95%: intervalos de confiança para 95%. Negrito: associação significativa (o IC95% passou de 1).

As variáveis associadas a cada uma das infecções analisadas, no modelo univariado, mas que não se mantiveram associadas no modelo múltiplo, foram: CT — “situação conjugal”, “uso de drogas nos últimos 12 meses” e “uso de drogas injetáveis na vida”; NG — “situação conjugal”, “trabalho sexual” e “uso de drogas nos últimos 12 meses”; e coinfecção — “trabalho sexual”.

## DISCUSSÃO

Considerando as estimativas de prevalências pontuais de infecções por CT e NG entre as mulheres trans e travestis, este estudo revelou que variaram entre as cidades e os sítios anatômicos de coleta, embora sem diferença estatisticamente significante. Os maiores valores foram para as infecções anorretais, seguidas das orofaríngeas e das uretrais. Quanto às características sociodemográficas, a idade (ser mais jovem) foi o único fator comum associado à maior ocorrência de CT, NG e coinfecção. Para CT, outras variáveis associadas foram “trabalho sexual” e “uso de drogas injetáveis na vida”; para NG, “uso de drogas injetáveis na vida”, assim como “orientação sexual”; e para coinfecção, “trabalho sexual”, além de “uso de drogas nos últimos 12 meses” — esta associada apenas a essa condição.

Chan et al.^10^ observaram, em revisão sistemática (1981 e 2015), nos EUA, que as prevalências das infecções extragenitais por CT e NG, em HSH, variaram a depender do sítio anatômico de coleta. Neste estudo, os valores observados para CT anorretal, envolvendo mulheres trans e travestis, foram semelhantes (8,5% *versus* 8,9%), porém a de CT orofaríngea foi o dobro da observada naquela revisão (3,2% *versus* 1,7%).

Callander et al.^20^, em estudo desenvolvido na Austrália (2010 a 2017), compararam as prevalências de infecção por CT e NG entre as populações trans (1.260) e cis — homens gays e bissexuais (387.848), atendidas em 46 clínicas de IST, e verificaram que as mulheres trans apresentaram chance 1,6 vez maior de adquiri-las. No presente estudo, verificou-se que, para CT, os valores foram semelhantes (11,5% *versus* 10,0%), porém para NG, a prevalência foi maior do que a observada naquele país (13,3% *versus* 8,6%).

Revisão sistemática conduzida por Van Gerwen et al.^11^, com população transexual, incluiu 25 estudos realizados em 11 países, incluindo o Brasil. Entre mulheres trans, verificaram-se as seguintes prevalências: CT — 2,7 a 24,7% e NG — 2,1 a 19,1%, com grande variabilidade segundo sítio anatômico. As prevalências globais obtidas no presente estudo apresentaram valores contidos nessas variações: 11,5% (IC95% 6,0–17,0) e 13,3% (IC95% 8,0–18,0), respectivamente.

Ao comparar os resultados do presente estudo com um conduzido no México^17^, com 206 HSH e seis mulheres trans, verificou-se que as prevalências de CT, segundo sítio anatômico, para ambas as populações investigadas, foram semelhantes para as orofaríngeas (3,2% *versus* 2,8%), menores para as anorretais (8,5% *versus* 11,8%) e para as uretrais (0,2% *versus* 7,5%), assim como para as infecções por NG — orofaríngeas (7,9% *versus* 7,5%), anorretais (8,7% *versus* 12,3%) e uretrais (0,7% *versus* 4,2%). Provavelmente, as prevalências encontradas nos sítios uretrais tenham sido maiores no estudo citado por ser majoritariamente constituído de HSH. A maioria das infecções por CT e NG foi assintomática em ambos os estudos, como observado na literatura^13^.

No Brasil, poucas pesquisas investigaram a prevalência de CT e NG em população trans, dificultando a comparação com o presente estudo. Em relação às populações diversas, os resultados foram maiores que os obtidos em parturientes^24^, em mulheres jovens sexualmente ativas^16,25^ e em mulheres com HIV^22^. Já estudo conduzido em seis cidades brasileiras, com 767 homens em clínicas de IST, observou prevalências superiores às deste estudo: CT (13,1% *versus* 11,5%) e NG (18,4% *versus* 13,3%)^21^.

Investigação em Salvador com 246 mulheres trans e travestis e HSH de 15 a 19 anos, de uma coorte – PrEP1519, entre 2019 e 2021, encontrou: 5,9% para infecção por CT (1,2% orofaríngea, 2,4% anorretal e 1,9% uretral); e 17,9% para infecção por NG (9,4% orofaríngea, 7,8% anorretal e 1,9% uretral)^19^, prevalências menores que as das amostras coletadas em Salvador no presente estudo — respectivamente, CT e NG: global (15,3 e 16,3%), orofaríngea (5,8 e 8,9%), anorretal (9,5 e 11,1%) e uretral (0,5 e 0,0%).

Estudo conduzido no Rio de Janeiro, com 391 HSH com e sem HIV, também observou prevalências maiores para CT do que as do presente estudo — CT anorretal (10,4% *versus* 8,5%) e uretral (2,2% *versus* 0,7%) — e menor para NG anorretal (2,5% *versus* 8,7%)^18^.

A prevalência de coinfecção deste estudo foi superior à verificada por Piazetta et al. para mulheres jovens no Sul do Brasil^25^ (3,6% *versus* 0,9%) e por Oliveira et al. para adolescentes HSH e trans em Salvador (3,6% *versus* 0,5%)^19^.

As variações de estimativas de prevalência dessas infecções nos diferentes estudos podem ser explicadas pelo envolvimento de populações com práticas sexuais diversas. Os estudos internacionais incluem mais usuários(as) de clínicas de IST, que provavelmente apresentam mais queixas e sintomas e maior prevalência de CT e NG que a população geral. Nesse sentido, o presente estudo se destaca por ter alcançado uma amostra robusta obtida, por meio de RDS, em cinco capitais, representativas das macrorregiões brasileiras, e apesar das limitações dessa técnica de recrutamento e estimativas possivelmente mais próximas das reais.

No presente estudo, embora não tenha sido observada diferença estatisticamente significativa entre as cidades, os valores das estimativas de prevalências pontuais apresentam grande variação, provavelmente decorrente das dificuldades de acesso e dos diferentes níveis de desigualdades sociais que marcam as macrorregiões brasileiras. As maiores prevalências pontuais observadas para a infecção por CT e a coinfecção foram em Salvador, e para a infecção por NG, em Manaus.

Entre os fatores associados a essas infecções, observados no presente estudo, com exceção do uso de drogas, a idade jovem, o trabalho sexual e o tipo de prática sexual também aparecem associados em outros estudos^2,3,12,17,21^.

Em uma revisão sistemática envolvendo estudos de IST realizados em diversos países, verificou-se que as prevalências para CT e NG nas mulheres trans apresentaram grande variabilidade. As maiores prevalências foram observadas em jovens com menos de 25 anos, minorias sexuais e de gênero, mulheres trans que fazem sexo com homens, negros e latinos^11^. O comportamento similar das variáveis associadas pode indicar que as diferenças estejam associadas a um menor poder estatístico, dada a menor quantidade de casos positivos para as duas infecções simultaneamente.

O método RDS tem sido amplamente utilizado em estudos com populações que seriam dificilmente recrutadas por outros métodos amostrais. No entanto, os resultados precisam ser interpretados com cautela, e podem ser representativos apenas das redes sociais eventualmente captadas pelo estudo, cujas características em geral são desconhecidas. Na ausência de melhores estratégias, o RDS continua a ser um método de eleição para estudos com populações de mulheres trans e travestis em vários contextos^31^.

O presente estudo contribui para atualizar a epidemiologia de IST entre mulheres trans e travestis e confirma que se apresentam em maior magnitude do que na população geral. Pelo fato de a maioria ser assintomática, seu controle continua a ser um desafio para a saúde pública, especialmente nas mais jovens, que exercem trabalho sexual e que fazem uso de drogas.

As mulheres trans e travestis constituem uma população vulnerabilizada e há barreiras que dificultam o seu acesso a serviços de saúde, demonstrando que a legislação existente não é suficiente para reverter esse quadro^32^. A alta vulnerabilidade desse grupo populacional pode estar associada à multiplicidade de parceiros sexuais e à violência sexual, culminando com risco aumentado às IST^33^. As mulheres trans e travestis deste estudo apresentaram características que as tornam ainda mais vulnerabilizadas, o que confirma a importância de considerá-las como um grupo prioritário para ações de prevenção e tratamento às IST investigadas.

Esses achados confirmam ainda a importância de incluir história detalhada das práticas sexuais nos atendimentos de rotina, especialmente para as populações de maior risco epidemiológico, além da disponibilização do rastreamento para detecção dessas infecções enquanto política pública de saúde. Tal recomendação já consta de diversos protocolos clínicos para IST, incluindo o brasileiro^15^.
